# Disability, distress and delayed access to care in functional neurological disorder: cross-sectional study from an Australian tertiary clinic

**DOI:** 10.1192/bjo.2026.11038

**Published:** 2026-05-13

**Authors:** Rebecca St L. Moss, Matthew J. Lennon, Sravan Anne, Emily Swift, Jessica W. Lo, Ishan C. Walpola, Michael H. Connors, Perminder S. Sachdev, Adith Mohan

**Affiliations:** Discipline of Psychiatry & Mental Health, UNSW Sydney, New South Wales, Australia; Neuropsychiatric Institute, Prince of Wales Hospital, Randwick, New South Wales, Australia; Centre for Healthy Brain Ageing, Faculty of Medicine and Health, UNSW Sydney, New South Wales, Australia

**Keywords:** Functional neurological disorder, neuropsychiatry, disability, quality of life

## Abstract

**Background:**

Functional neurological disorder (FND) is a common condition, but there remain substantial gaps in our understanding of its effects, particularly on severity of disability and health status.

**Aims:**

To characterise disability, quality of life and psychological and somatic symptom comorbidity in individuals with FND attending a specialist multidisciplinary clinic in Australia.

**Method:**

We conducted a cross-sectional analysis of patients assessed at an FND clinic in Sydney, Australia, between August 2022 and February 2025. We assessed disability (World Health Organization Disability Assessment Schedule (WHODAS 2.0)), health-related quality of life (EQ-5D five-level version (EQ-5D-5L), 36-Item Short Form Health Survey), psychological distress (Depression Anxiety and Stress Scale-21, Kessler Psychological Distress Scale (K10)) and somatic symptom severity (Patient Health Questionnaire-15 (PHQ-15)).

**Results:**

The cohort (*N* = 105) was predominantly female (74.3%), with a mean age of 35.4 (s.d. = 13.3) years. Functional seizures (46.7%) and functional weakness (45.7%) were the most common presentations. Only 33.7% were employed; 42.9% were unable to work because of FND. The average delay from diagnosis to clinic assessment was 356 days (s.d. = 463). WHODAS 2.0 scores indicated high levels of disability, exceeding international norms for both physical and mental illness. EQ-5D-5L scores were low, with 15.8% reporting a health state rated as ‘worse than death’. Psychological distress and somatic symptom severity were high: 49.5% scored in the K10 ‘very high’ range and 54.5% had high PHQ-15 scores.

**Conclusions:**

FND is associated with significant functional disability, poor quality of life and high levels of psychological and somatic symptom comorbidity. Delays in accessing appropriate care and high rates of vocational disruption highlight the need for earlier diagnosis and better access to integrated, multidisciplinary FND services in Australia.

Functional neurological disorder (FND) is a neuropsychiatric disorder commonly presenting with motor symptoms (functional weakness or movement disorder) and functional seizures, although symptoms may include cognitive difficulties, dizziness, speech and swallowing difficulties, visual disturbances or sensory deficits. Diagnosis has traditionally relied on exclusion or incongruence with other neurological disorders, but a contemporary framework focuses on identifying typical or positive clinical signs and internal inconsistency.^1,2^ FND has a prevalence of 80–140 per 100 000 persons and an annual incidence of 10–22 per 100 000 persons.^1^ It is three times more common in women than in men,^3,4^ and is more prevalent among individuals from lower socioeconomic backgrounds.^5,6^

FND is highly prevalent in neurological settings, comprising 16% of out-patient referrals^7,8^ and 9% of in-patient admissions.^9^ However, there are often delays in FND diagnosis because of, in part, diagnostic uncertainty, historical stigma, low clinician confidence and a lack of dedicated specialist services.^10,11^ A 2023 Italian study reported a median diagnostic delay of 4 years for FND, compared with 1 year for neurological disorders generally.^12^ Such delays are consistently associated with increased healthcare costs and poorer outcomes.^13^

Despite high healthcare utilisation, clinical outcomes remain poor. Health utility scores, where 1 indicates full health and 0 the worst health imaginable, have previously been generated in cohorts of people with functional motor symptoms (0.40)^14^ and functional seizures (0.53);^15^ suggesting a significant negative impact on health and well-being. However, there remain substantial gaps in data available on disability and functioning in FND. There are no quantitative studies focusing on the severity and forms of disability associated with FND, with no focused reporting on disability since a 2011 cohort of people with neurological symptoms unexplained by organic disease.^13^ Furthermore, there is minimal information on the impact of FND on ability to engage with work and study, despite the preponderance of this disorder in young and middle-aged adults.^16,17^

As of yet, no comprehensive clinical characterisation has been published in an Australian cohort,^10,18,19^ although two studies have examined demographics and healthcare utilisation.^7,20^ This study presents a detailed cross-sectional analysis of patients assessed at an Australian public specialist FND clinic. We describe patient demographics, symptom profiles, employment status, disability, psychological distress and quality of life, with benchmarking against population norms and international FND cohorts where possible.

## Method

### Participants

Adult patients assessed at the Mindgardens Functional Neurological Disorders Clinic at Prince of Wales Hospital, Sydney, between August 2022 and February 2025 were included. The clinic was established under the governance framework of a tertiary, adult neuropsychiatry service in the New South Wales (NSW) public mental health system and operated within the scope of this service. As such, specific exclusions outside the operational scope of the unit were unavoidable and included patients aged >65 years and those with acute substance-related disorders and acute psychiatric risks requiring urgent attention through another secondary care service. FND referrals were accepted regardless of symptom type and duration or severity of illness, and we included referrals from both regional and metropolitan areas. The full inclusion and exclusion criteria for clinic participation are outlined in outlined in Box 1.

Box 1 Inclusion and exclusion criteria for referral to the Mindgardens Functional Neurological Disorders Clinic

Inclusion criteria

- Aged 18–65 years
- FND diagnosed by a specialist neurologist
- Confirmation that that the diagnosis has been explicitly discussed with the patient before referral to the FND clinic
- Commitment by referring specialist to stay involved in patient’s care after clinic interventions are completed
- All FND-related investigations have been completed
- Patient consenting and aware of referral to clinic

Exclusion criteria

- Active drug and/or alcohol issues requiring treatment
- Active acute psychiatric disorder that needs urgent treatment
- Major neurocognitive disorder or developmental disability that precludes informed consent
- Active medicolegal or worker’s compensation issues
- Acute psychiatric or behavioural risk issues (e.g. acute suicidality, aggression risk)
- Chronic pain and/or fatigue is predominant clinical presentation

FND, functional neurological disorder.

Demographic and clinical information collected included age, gender, time from FND diagnosis to clinic assessment, and acceptance of the diagnosis. Primary neurological symptoms and associated symptoms (e.g. pain, cognitive fog) were recorded from referral forms, along with healthcare utilisation data, including emergency department presentations and in-patient hospital admissions as reported by referring specialists and patients. Allied health involvement at the time of referral was documented. Employment and study status were noted, including reasons for not working or studying, and the duration of this absence if attributed to FND. Socioeconomic status was assigned based on patients’ residential postcodes, using the 2021 Socioeconomic Indexes for Areas data from the Australian Bureau of Statistics.^21^

### Measures

Participants completed a battery of validated measures as part of their baseline clinic assessment. This included six self-report questionnaires administered before the initial consultation and two clinician-rated scales completed immediately after assessment. On average, self-reported measures took 30–45 min to complete and were administered in two blocks, the first being sent out to the patient prior to attendance at the clinic, and the second on arrival before assessment. The clinician-rated scales took 5–10 min to complete. The self-reported measures included the World Health Organization Disability Assessment Schedule (WHODAS 2.0),^22^ the Depression Anxiety and Stress Scale (DASS-21),^23^ Kessler Psychological Distress Scale (K10),^24^ Patient Health Questionnaire-15 (PHQ-15),^25^ EQ-5D five-level version (EQ-5D-5L)^26^ and the 36-Item Short-Form Health Survey (SF-36).^27^ The SF-36 is the most widely used measure of illness impact in FND intervention studies,^28^ although it is primarily conceptualised as a measure of health-related quality of life.^29^ The clinician-rated scales included the Clinical Global Impression – Severity Scale (CGI-S)^30^ and the Health of the Nation Outcome Scales (HoNOS).^31^ Further details on these scales and their scoring are provided in Table 1.

**Table 1:** Summary of self-report and clinician-rated measures

Measure	Description
World Health Organization Disability Assessment Schedule 2.0	A 36-item instrument based on the biopsychosocial model of disability and functioning. It assesses difficulties across six domains: cognition, mobility, self-care, getting along with others, life activities (household and work/study) and societal participation. Domain and total scores were calculated using the complex scoring method, with results scaled from 0 (no disability) to 100 (complete disability). Scores were interpreted with reference to data from international treatment groups and the general population taken from Üstün et al^22^
Depression Anxiety and Stress Scale-21	A 21-item self-report instrument designed to assess symptoms across three domains: depression, anxiety and stress. Each subscale consists of seven items rated on a four-point Likert scale ranging from 0 (‘Never’) to 3 (‘Almost always’). Details of scoring are included in the Supplementary Methods
Kessler Psychological Distress Scale	A ten-item self-report measure assessing general psychological distress over the past 4 weeks. Total scores ranging from 10 (no distress) to 50 (severe distress), and scores are classified into four severity bands: low (10–15), moderate (16–21), high (22–29) and very high (30–50)
Patient Health Questionnaire–15	A 15-item self-report measure assessing somatic symptom severity over the past 4 weeks. Total scores range from 0 to 30 for females, and up to 28 for males (as the menstrual problems item is excluded). Total scores can represent low (5-9), medium (10-14) and high somatic symptom severity (≥15)
EQ-5D, five-level version	A widely used measure of health-related quality of life, it includes a single visual analogue scale measuring overall current health (where 100 is the best health imaginable), alongside five dimensions (mobility, self-care, activity, pain and anxiety/depression). Each domain is rated on five levels of severity, from no problems to extreme problems/unable. A utility index score was calculated using an Australian-specific value set. More details on utility score calculations are provided in the Supplementary Methods
36-Item Short-Form Health Survey	A self-report survey of health-related quality of life, used to assess health across eight domains: physical functioning, bodily pain, role limitations owing to physical health, role limitations owing to emotional problems, emotional well-being, social functioning, energy/fatigue and general health perceptions. Domain scores are standardised on a 0–100 scale, with lower scores indicating a less favourable health state
Clinical Global Impression – Severity Scale	A clinician-rated measure of overall illness severity, rated on a seven-point scale from 1 (normal, not at all ill) to 7 (among the most extremely ill)
Health of the Nation Outcome Scales	A 12-item clinician-rated instrument used to assess psychological symptoms, behavioural difficulties, impairment and social functioning in individuals with mental health conditions. Each item is rated on a five-point scale (0–4), with higher scores indicating greater severity

### Data analysis

This study aimed to describe the demographic, clinical and functional characteristics of a cohort of patients with FND, including presenting symptoms, disease burden and quality of life, as part of a clinic evaluation study. As the objective was primarily descriptive, minimal statistical analyses were employed. Given established differences in FND prevalence by gender (female predominance) and age (typically peaking in the late 30s to early 40s), comparisons were conducted between gender groups and across age groups. For age-based analyses, participants were divided into three approximately equal tertiles: 17–25 years, 26–40 years and 41 years and older.

Continuous variables are reported as means with standard deviations, and categorical variables as counts with corresponding percentages. Between-group comparisons of continuous variables were conducted using analysis of variance, whereas chi-squared (*χ* ^2^) tests were used for categorical data. All analyses were performed using R statistical software (version 4.4.2). Given the exploratory and descriptive nature of the study, statistical significance was defined as *p* < 0.05, with no adjustments made for multiple comparisons.

### Ethics

The procedures involving human patients were assessed by the South Eastern Sydney Local Health District Human Research Ethics Committee (HREC) and deemed not to require further formal HREC review (approval number 2022/ETH00904) as a quality improvement/quality assurance activity in accordance with NSW Health guidelines. The need to obtain informed consent from participants was also waived by the HREC. Data collected was stored on secure NSW Health servers and deidentified for aggregate analyses using unique random identifiers delinked from clinical identifiers, as per the protocol reviewed by the HREC.

## Results

### Demographic and clinical characteristics

A total of 105 participants were assessed at the clinic between August 2023 and February 2025, with a mean age of 35.4 years (s.d. = 13.3, range: 17–66), and 74.3% of participants were female (Table 2). At the time of assessment, 33.7% of participants were employed and 11.2% were enrolled in academic study. Among those not working (52 participants), the majority (86.5%) identified their FND as the primary reason for unemployment, with a mean duration of 648.6 days (approximately 1.8 years) since last employment.

**Table 2:** Demographic and clinical characteristics of participants with functional neurological disorder (*N* = 105)

Demographics	
Mean age, years (s.d.)	35.4 (13.3)
Gender (female, %)	74.3
Mean postcode socioeconomic deciles (s.d.)	7.1 (2.4)
Currently working, *n* (%)	
Yes	33 (33.7)
No	52 (53.1)
Not applicable	13 (13.3)
Currently studying, *n* (%)	
Yes	11 (11.2)
No	17 (17.3)
Not applicable	70 (71.4)
Reason not working because of FND, *n* (%)	45 (42.9)
Allied health involvement at referral, *n* (%)	
Psychologist	50 (47.6)
Occupational therapist	7 (6.7)
Physiotherapist	26 (24.8)
Speech pathologist	3 (2.9)
Diagnosis and hospital presentations, mean (s.d.)	
Days between diagnosis and baseline assessment	356.6 (463)
Days between referral and baseline	110.4 (76.6)
Days between diagnosis and referral	251.4 (452.3)
Emergency presentations, *n* (%)	
0	34 (32.4)
1	28 (26.7)
2	22 (21)
3 or more	21 (20)
Hospital admissions, *n* (%)	
0	41 (39)
1	38 (36.2)
2	19 (18.1)
3 or more	7 (6.7)
Primary FND symptoms at referral, *n* (%)	
Non-epileptic seizures or attacks	49 (46.7)
Weakness or paralysis	48 (45.7)
Abnormal movement	43 (41)
Speech symptom	20 (19)
Sensory disturbance	26 (24.8)
Other	19 (18.1)

FND, functional neurological disorder.

For the ‘currently working’ and ‘currently studying’ variables, responses were marked as not applicable if the participant was not studying or working to begin with (i.e. they had never planned to study or work). Other primary symptoms include balance, gait, swallowing and fainting episodes, among others.

Participants reported an average delay of 356 days between receiving a diagnosis of FND and their initial clinic assessment. This included a mean of 251 days between diagnosis and referral, and 110 days between referral and baseline assessment.

The most common primary symptoms as reported by the referring specialist were functional seizures (46.7%), weakness or paralysis (45.7%) and abnormal movements (41%), followed by speech disturbances (24.8%) and sensory symptoms (19%). Associated overlapping symptoms reported by referrers included fatigue (45.7%), cognitive difficulties (39%), pain (30.5%) and gastrointestinal complaints (5.7%).

Healthcare utilisation was high: 67.6% of participants had presented to an emergency department at least once for FND-related symptoms, with 20% attending three or more times. Additionally, 61% had experienced at least one hospital admission. At the time of assessment, 47.6% were engaged with a psychologist, 24.8% with a physiotherapist, 6.7% with an occupational therapist and 2.9% with a speech pathologist.

### Clinical profile at baseline

Participants reported high levels of disability, somatic symptom burden and psychological distress across multiple domains (Supplementary Table 1 available at https://doi.org/10.1192/bjo.2026.11038).

**Fig. 1:**
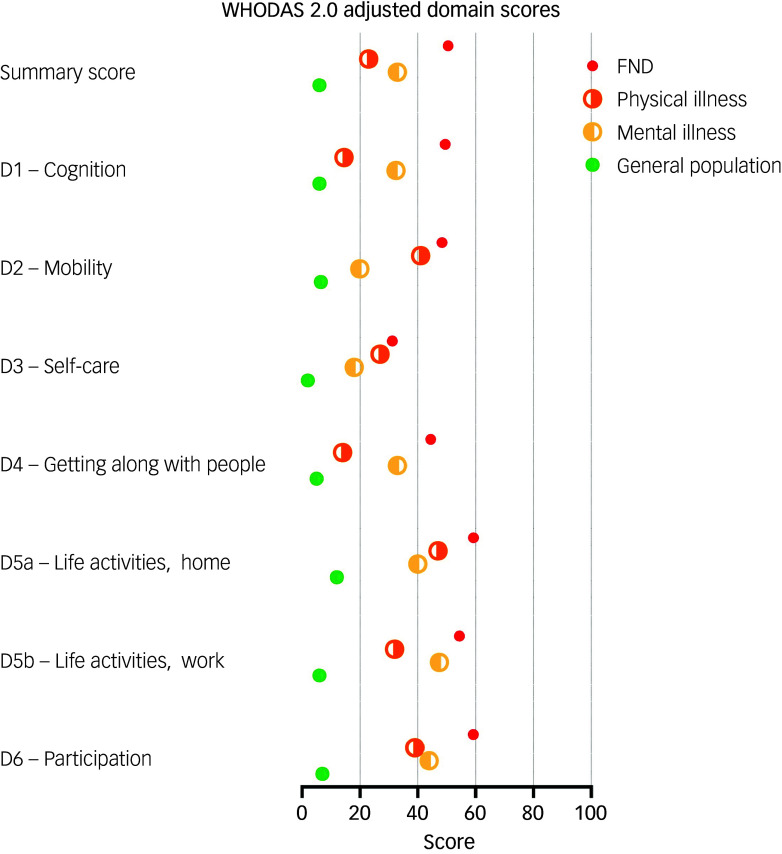
World Health Organization Disability Assessment Schedule 2.0 (WHODAS 2.0) summary and domain scores for this cohort of people with functional neurological disorder (FND), compared with population norms from Üstün et al.^22^ Higher scores reflect greater disability (range: 0–100). D1–D6: domain 1 to domain 6.

The mean WHODAS 2.0 summary score was 50.5 (s.d. = 22.6), indicating a moderate to high level of disability that exceeded international norms for physical illness, mental illness and the general population (Fig. 1). The most affected domains were participation in society (mean: 59.2, s.d. = 23.9), life activities at home (mean: 59.3, s.d. = 33.6), and life activities at work or school (mean: 54.4, s.d. = 32). Cognition (mean: 49.5, s.d. = 24.7) and mobility (mean: 48.4, s.d. = 29.1) were also significantly affected. On average, participants reported being affected by their condition on 22.1 days over the preceding month, with complete inability to perform duties on 13.2 days and needing to reduce activity on 15.9 days.

**Fig. 2:**
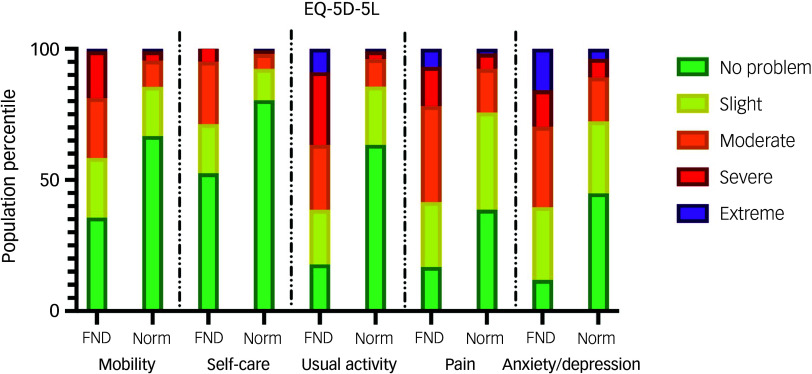
Frequencies of problems reported across the five EQ-5D five-level (EQ-5D-5L) domains in this cohort of individuals with functional neurological disorder (FND), compared with Australian general population norms (‘Norm’) from Redwood et al.^42^

Health-related quality of life was markedly low. On the EQ-5D-5L, anxiety/depression, pain and usual activities were the most affected domains, with only 11.9, 16.8 and 17.8% of participants reporting no issues, respectively. In contrast, personal care was relatively preserved, with 52.5% reporting no difficulty. Across all five dimensions, patients were more likely to have moderate (22.8–36.6%), severe (5.0–27.7%) or extreme problems (0–15.8%) than the general population (Fig. 2), apart from extreme problems in self-care (0% in cohort versus 0.6% in norms). The mean health utility score derived from the EQ-5D-5L was 0.35 (s.d. = 0.37). Notably, 15.8% of the cohort had a utility score of 0 or below; equivalent to a health state perceived as worse than death by general population standards. Patients rated their health status at an average of 49.9 (s.d. = 23.2) on the EQ-5D visual analogue scale. The SF-36 further reflected poor functioning, with especially low scores in the domains of role limitations owing to physical health (mean: 12.6, s.d. = 28.9), energy/fatigue (mean: 26.4, s.d. = 21.2) and emotional role limitations (mean: 30.4, s.d. = 40.6). Social functioning (mean: 33.0, s.d. = 29.4) and general health (mean: 33.5, s.d. = 20.7) were also considerably reduced.

**Fig. 3:**
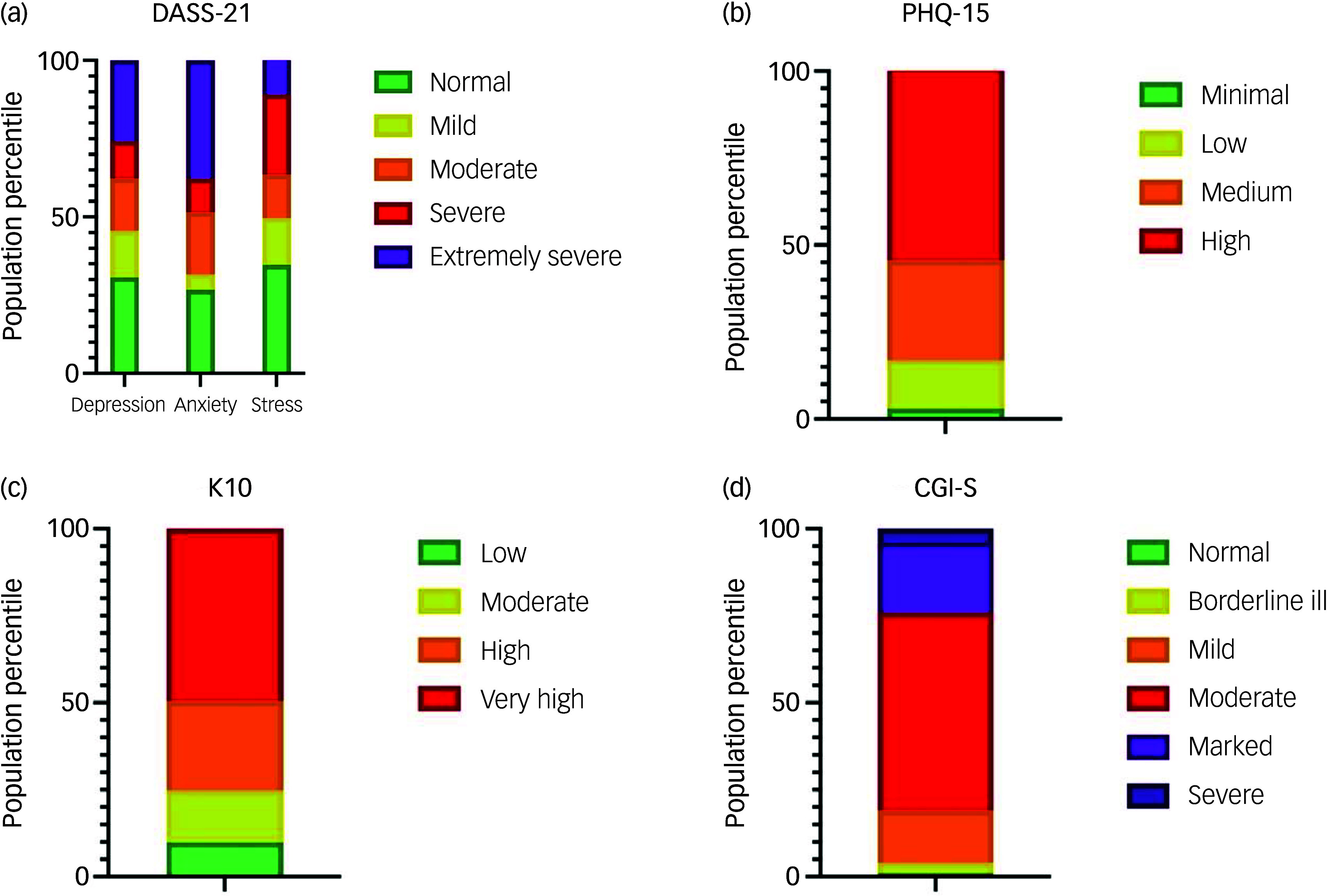
Proportions of participants in each severity category across different instruments. (a) DASS-21: Depression, Anxiety and Stress Scale (21-item); (b) PHQ-15: Patient Health Questionnaire-15 (somatic symptom severity); (c) K10: Kessler Psychological Distress Scale; (d) CGI-S: Clinical Global Impression Scale – Severity. Each panel displays the distribution of participants across standard severity classifications for that measure.

Participants self-reported a considerable burden of psychological distress. On the DASS-21 (Fig. 3(a)), a majority of patients reported experiencing moderate to extremely severe anxiety (68.3%) depression (54.4%) and stress (50.5%). K10 scores (Fig. 3(c)) also indicated severe psychological distress, with nearly half (49.5%) in the ‘very high’ range and only 9.9% in the ‘low’ range. The PHQ-15 (Fig. 3(b)) showed that 54.5% of participants experienced high somatic symptom severity, with only 3% reporting minimal somatic symptom burden. Notably, 93.1% of participants reported being bothered (a lot or a little) by fatigue, followed by 87.1% bothered by trouble sleeping, 86.1% by headaches, 84.2% with limb/joint pain, 80.2% by back pain and 77.2% by dizziness (Supplementary Table 2)

Clinician-rated assessments were reasonably consistent with these findings, albeit with patients seeming to self-rate as being marginally worse than clinician estimations. On the CGI-S (Fig. 3(d)), most participants were rated as moderately (57%) or markedly ill (20%), with an average score of 4.0 (s.d. = 0.9). The HoNOS revealed moderate impairment, with a mean score of 10.3 (s.d. = 4.5).

### Differences by gender and age groups

Male participants in the cohort were significantly older than female participants (mean age: 41.0 (s.d. = 11.9) *v*. 33.5 (s.d. = 13.3); *p* = 0.011). Women were more likely to have presented to the emergency department, with 66.9% having at least one presentation compared with 40.7% of men (*p* = 0.003). Men reported significantly lower health utility scores than females (mean: 0.29 (s.d. = 0.42) *v*. 0.37 (s.d. = 0.34); *p* = 0.024) (Supplementary Tables 3 and 4).

When comparing age tertiles (17–25, 26–40 and ≥41 years), several statistically significant differences emerged (Supplementary Tables 5 and 6). Older participants had significantly longer durations of unemployment than younger participants (mean days since last working: 226.8 (s.d. = 142.6), 996.0 (s.d. = 1329.4) and 1896.6 (s.d. = 2801.8), for 17–25, 26–40 and ≥41 years, respectively; *p* = 0.025). Symptom presentation also varied by age: functional seizures were more common in the youngest tertile (69.0%) than in the middle (45.2%) or oldest (29.4%) groups (*p* = 0.007), whereas cognitive symptoms were reported more frequently by older participants (17–25 years: 24.1%, 26–40 years: 35.7%, ≥41 years: 55.9%; *p* = 0.031). Additionally, emergency department presentations were less frequent with increasing age (one or more presentations: 93.1, 66.7 and 47.1%, for 17–25, 26–40 and ≥41 years, respectively; *p* = 0.001). Age-related differences were also evident in somatic symptoms and health-related quality of life. The youngest group had significantly higher PHQ-15 scores (mean: 17.4, s.d. = 5.2) than the middle (mean: 14.8, s.d. = 6.4) and oldest (mean: 13.1, s.d. = 5.0) groups (*p* = 0.005). They also reported lower SF-36 scores in the energy/fatigue domain (mean: 21.2 (s.d. = 17.1), 24.9 (s.d. = 23.9) and 33.0 (s.d. = 19.5), for 17–25, 26–40 and ≥41 years, respectively; *p* = 0.03) and the general health domain (mean: 27.5 (s.d. = 18.4), 32.2 (s.d. = 20.1) and 40.5 (s.d. = 22.0), for 17–25, 26–40 and ≥41 years, respectively; *p* = 0.014), indicating poorer subjective well-being and vitality.

## Discussion

This study offers, for the first time, a comprehensive, cross-sectional characterisation of Australian patients with FND attending a multidisciplinary tertiary clinic. The cohort composition was broadly similar to other representative international FND cohorts. Most notably, there was a clear predominance of female participants (approximately 3:1), consistent with global literature demonstrating that FND is three times more prevalent in women than men.^1,16^ This consistent gender ratio across clinical and research settings supports the generalisability of our findings and reflects likely biopsychosocial contributors, including gender-based endocrinological factors,^32^ differential exposure to trauma, coping responses, help-seeking behaviours and clinician bias.^4^ The symptom profiles reported were also closely aligned with other large studies,^1,17^ with functional seizures (46.7%), weakness/paralysis (45.7%) and abnormal movements (41%) were the most commonly reported primary symptoms in our cohort.

Patterns across age groups suggested the potential for different symptom subtypes across age groups. Numerous aetiological and precipitating factors in FND vary in their likelihood between life stages, such as exposure to complex trauma in childhood or the onset of comorbid neurological disorders (e.g. epilepsy in childhood, Parkinson’s disease and dementia in older age). Functional neurological symptoms can be influenced by illness behaviour modelled by peers and relatives, and they may be driven by cultural models of illness and social stigma that determine what kinds of illnesses are ‘valid‘ or ‘deserving of care’.^33^ This study from a cohort of working-age adults excludes children and the elderly; however, patterns corresponded with previous findings: non-epileptic seizures were more common in younger participants,^34^ whereas cognitive symptoms were more frequently reported in older individuals. A pattern of episodic or paroxysmal symptoms (such as functional seizures) in younger adults may account for the increased rates of emergency department presentations in our younger age tertiles. Incidence patterns of different functional neurological symptoms across the lifespan remain poorly characterised, partly secondary to many studies grounded in age-specific settings, such as functional seizures in children’s hospitals or cohorts of functional cognitive disorder studied within memory clinics for older adults. These gaps in the literature suggest a role for future collaborative research investigating FND across all subtypes and across the lifespan.

### Substantial disability and vocational disruption

To date, no studies to our knowledge have characterised disability and functioning in FND by using the WHODAS 2.0, making this cohort a novel contribution to the international literature. The mean summary score of 50.5 reflects a high level of disability, placing participants just above the 95th percentile of the general population (mean: 6.0) and well above international norms for physical (mean: 23.0) and mental illness (mean: 33.0).^35^ This level of impairment exceeds that reported for many serious neurological and psychiatric conditions, including stroke (mean: 49.8), multiple sclerosis (mean: 41.0), schizophrenia (mean: 39.5) and major depression (mean: 41.6).^36–38^ Disability was particularly marked in domains related to societal participation (mean: 59.2), domestic and occupational life (mean for home: 59.3, mean for work/study: 54.4), cognition (mean: 49.5) and mobility (mean: 48.4), reflecting the broad, multidimensional impact of the condition. Even among participants who were not employed – and thus excluded from work-specific WHODAS 2.0 items – substantial limitations were observed across all other domains.

This high level of disability was reflected in widespread vocational disengagement. Only 33.7% of participants were employed and just 11.2% were enrolled in study, despite the cohort being predominantly of working age. These findings align with previous Australian survey data, where 70% of individuals with FND reported being unable to work or study because of their symptoms.^18^ Our data newly indicate that an overwhelming majority (86.5%) attributed their unemployment specifically to FND, with an average time out of work of 1.8 years.

Taken together, these findings affirm that FND and its associated symptoms (including brain fog, fatigue, pain and distress) are associated with severe functional disability. This highlights the urgent need for early, recovery-oriented care that promotes re-engagement in meaningful roles, including employment, education and social participation.

### Barriers to timely and comprehensive care

A striking feature of this cohort was the substantial delay between diagnosis and access to specialised multidisciplinary care. This expands on existing knowledge of a substantial treatment gap for patients with functional seizures.^39^ On average, 356 days elapsed between receiving a diagnosis of FND and the initial clinic assessment, with over 250 of those days occurring before referral. Crucially, these delays occurred despite participants already having a confirmed diagnosis from a neurologist. A subset of patients were diagnosed with FND before the establishment of this new clinic, and their treating neurologist may have had initial difficulties in identifying a suitable clinician with expertise in FND. However, such delays continued over time despite an established referral pathway and increased awareness about this service. A previous survey of FND services in Australian and New Zealand clinics identified limited capacity, access to allied health support, insufficient funding, large service demand, complex patients and poor discharge supports, particularly in remote areas, as the core challenges in their service provision.^10^ These issues may also contribute to prolonged wait times.

Although these systemic issues are a major factor in treatment delays, the literature on provider attitudes and patient-level psychological factors offers additional insights. Clinicians can hold implicit and explicit biases against the legitimacy of FND and its treatment, which negatively correlates with treatment optimism and their likelihood of referring patients for specialist interventions.^40^ Patient-level factors such as illness severity, psychosocial disability, functional impairment, economic hardship and employment instability can further delay engagement with services, disproportionately affecting those in lower socioeconomic circumstances and regional settings.^41^

Most patients had had no specialist FND interventions despite high healthcare utilisation in the acute setting; with over two-thirds presenting to emergency departments for FND-related symptoms, and 61% having had at least one hospital admission. Many participants had had no treatment for their symptoms before attending the clinic highlighting the paucity of public sector services for FND in NSW. Fewer than half were seeing a psychologist or physiotherapist, and even fewer were engaged with occupational therapists or speech pathologists. These findings align with previous studies^9^ and suggest that patients often cycle through acute care settings without receiving diagnosis-specific treatment.

Together, these results suggest that there are barriers in the post-diagnostic phase – reflecting limited clinician awareness of treatment options, system fragmentation and inconsistent referral practices. As earlier intervention is strongly associated with better outcomes in FND,^14,15^ the difficulty accessing timely, coordinated multidisciplinary care may contribute to preventable distress, disability and repeated acute service use.

### Compromised quality of life and severity of psychological distress

Health-related quality of life was profoundly impaired in this cohort. The mean EQ-5D-5L utility score was 0.35 – substantially lower than the Australian population norm of 0.86^42^ and below utility values typically observed in most chronic diseases. A recent systematic review^43^ reported mean utility scores of 0.83 for diabetes, 0.77 for cardiovascular disease, 0.75 for chronic obstructive pulmonary disorder and 0.56 for multiple sclerosis. Only patients with advanced cancer or end-stage renal disease had comparable health-related quality-of-life levels. Notably, 15.8% of participants in our sample reported utility scores of 0 or below, reflecting a perceived health state worse than death according to general population value sets. These findings underscore the profound perceived burden of FND and highlight its parity – if not increased severity – when compared with other serious health conditions. Given the widespread use of EQ-5D utility scores in economic evaluations and healthcare resource allocation, these data provide justification for greater investment in FND-specific services, interventions and workforce development.

The burden of FND is further illustrated by psychological distress and somatic symptom data. The exclusion of patients with acute psychiatric risk or active substance use may have negatively biased our estimates of distress in people with FND. However, most participants fell within the moderate, severe or extremely severe ranges on the DASS-21 for depression, anxiety and stress, and nearly half (49.5%) scored in the very high range on the K10. These findings are support established associations between FND and stress, anxiety and depression.^44–46^

There was a striking difference between the severity and frequency of somatic symptoms reported by participants and their referrers. A majority of the sample (54.5%) reported high somatic symptom severity on the PHQ-15, with only 3% reporting minimal somatic symptoms. Where 93.1% of participants self-reported fatigue on the PHQ-15, referring clinicians noted fatigue in only 45.7%. This finding points to possible oversight by referring clinicians in our study. Our findings of somatic symptom severity are aligned with a European survey of 1048 patients with FND^47^ (mean age: 42 years, 86% female), which found that participants on average had 9.9 FND symptoms (primary and associated), with the most common in fact being fatigue (93%), memory difficulties (80%) and headache (70%).

Interestingly, even within the FND clinic, patients generally rated themselves as more severely ill than the clinician rating. For example, the average CGI-S was 4.0 (moderately ill), whereas 49.5% of K10 scores were very high and 54.5% of PHQ scores were in the high range. In comparison, a study of 100 patients with depression^48^ found that discrepancies between clinician-rated and self-rated scales were most prominent in those with childhood trauma, neurodiversity (i.e. autistic traits) and maladaptive coping styles, all of which are prevalent in FND cohorts.

### Limitations

This study has several limitations. First, its cross-sectional design prevents examination of temporal relationships between disability, psychological symptoms and vocational disengagement. Longitudinal studies are needed to clarify these dynamics over time. Second, there are limits to the generalisability of our sample. It may be unrepresentative given that it is taken from a single centre in a large metropolitan setting. As a tertiary clinic, there may be a referral bias toward greater severity or chronicity, and noting the prolonged duration between diagnosis and referral, our sample may have excluded those who had a rapid remission after diagnosis or early access to treatment and were thus never referred to our service. The requirement for a confirmed neurologist diagnosis likely overrepresented motor and seizure subtypes, underrepresenting other FND presentations such as functional speech or swallowing disturbances, as patients presenting with these symptoms may come to the attention of non-neurology specialists. The clinic’s close ties to an epilepsy service may have further skewed the sample toward functional seizures. There were also limitations in our data on cohort characteristics. Although we screened for psychiatric and neurological comorbidities during clinical care, comprehensive lists of diagnoses were not recorded. Data from referrers was focused on identifying any high acuity comorbidities or other disorders that would be most appropriate for referral to other local services. We did not routinely gather information on gender and sexual identities, and thus cannot contribute to the growing literature on FND in sexual and gender minorities.^49^

Furthermore, although one of the larger Australian FND cohorts, the sample was underpowered for some subgroup analyses – particularly by symptom subtype or treatment engagement. Comparisons by age and gender should be interpreted with caution because of small subgroup sizes and risk of type 1 error. Finally, the WHODAS 2.0 excludes work-related items for unemployed participants, potentially underestimating the vocational impact of FND. Future research should include targeted measures of occupational functioning and longitudinal follow-up.

In conclusion, this study provides a comprehensive clinical characterisation of an Australian cohort of people with FND attending a specialist multidisciplinary clinic. Patients reported profound functional disability, diminished quality of life and high rates of psychological and somatic symptom burden. Most were of working age, yet unemployed or not studying because of their condition, reflecting significant vocational impairment. Long delays in accessing care, high emergency and in-patient service use, and limited multidisciplinary engagement highlight systemic gaps in service delivery. These findings underscore the urgent need for earlier diagnosis, improved care pathways and broader access to integrated, FND-specific services across Australia.

## Supporting information

10.1192/bjo.2026.11038.sm001 Moss et al. supplementary material Moss et al. supplementary material

## Data Availability

The data are not publicly available due to containing information that may compromise the privacy of research participants.

## Supplementary material

The supplementary material is available online at https://doi.org/10.1192/bjo.2026.11038
