# Supplementary material for: Disability, distress and delayed access to care in functional neurological disorder: cross-sectional study from an Australian tertiary clinic
Source: BJPsych Open. 2026 May 13;12(3):e128. doi: 10.1192/bjo.2026.11038 (PMC13169049; doi:10.1192/bjo.2026.11038)
Supplement: Moss et al. supplementary material [file S2056472426110382sup001.docx]

**Supplementary Material**

**Disability, Distress, and Delayed Access to Care in Functional Neurological Disorder: A Cross-Sectional Study from an Australian Tertiary Clinic**

Table of Contents

[Supplementary Methods 2](#_Toc204942441)

[Supplementary Table 1: Self report and clinician rated measures at baseline 4](#_Toc204942442)

[Supplementary Table 2: Somatic symptom severity - individual items of the PHQ-15 6](#_Toc204942443)

[Supplementary Table 3: Comparison of baseline characteristics of male and female participants 7](#_Toc204942444)

[Supplementary Table 4: Comparison of baseline outcomes of males and females 8](#_Toc204942445)

[Supplementary Table 5: Comparison of baseline characteristics of the three age tertiles 10](#_Toc204942446)

[Supplementary Table 6: Comparison of baseline outcomes of the age tertiles 11](#_Toc204942447)

[Supplementary References 13](#_Toc204942448)

## **Supplementary Methods**

1. **WHODAS 2.0 scores in international treatment groups**

The following treatment group norms for WHODAS 2.0^1^ are included in figure 1 within the paper.

|  | **Mental health problems** | **Physical health problems** | **General population** |
| --- | --- | --- | --- |
| **Summary Score** | 33 | 23.0 | 6 |
| **Domain 1** | 32.5 | 14.5 | 6 |
| **Domain 2** | 20 | 41.0 | 6.5 |
| **Domain 3** | 18 | 27.0 | 2 |
| **Domain 4** | 33 | 14.0 | 5 |
| **Domain 5a** | 40 | 47.0 | 12 |
| **Domain 5b** | 47.5 | 32.0 | 6 |
| **Domain 6** | 44 | 39.0 | 7 |

1. **Australian Population Norms of EQ-5D-5L from Redwood et al. (2024)**

The following norms for EQ-5D-5L^2^ are included in figure 3 within the paper.

|  | **No Problem** | **Slight** | **Moderate** | **Severe** | **Extreme/Unable** |
| --- | --- | --- | --- | --- | --- |
| **Mobility** | 66.7 | 18.8 | 10 | 3.5 | 1 |
| **Self-Care** | 80.4 | 12 | 5.6 | 1.4 | 0.6 |
| **Usual Activities** | 63.4 | 22.2 | 10.5 | 2.9 | 1 |
| **Pain/Discomfort** | 38.6 | 37.1 | 16.7 | 5.8 | 1.8 |
| **Anxiety and Depression** | 44.9 | 27.5 | 16.7 | 7.1 | 3.8 |

1. **Computation of EQ-5D-5L Utility Score**

The EQ-5D is a generic health questionnaire that assesses health-related quality of life across five dimensions: mobility, self-care, usual activities, pain/discomfort, and anxiety/depression. The utility value represents the population represents the population's preference for that health state, ranging from 0 (death) to 1 (full health). Such utility values can be calculated using a value set or weights, specific to a country or population, that has been calculated using discrete choice experiments.

In our study we have used the Australian discrete choice experiment from the following paper:

“Norman R, Mulhern B, Lancsar E, et al. (2023) The use of a discrete choice experiment including both duration and dead for the development of an EQ ‑ 5D ‑ 5L Value Set for Australia method for the economic evaluation of new pharmaceuticals. *PharmacoEconomics* 41(4): 427–438.” ^3^

Based on their results we used the weights from their model 4. The R script, including weights

for each health states, used to compute the utility score is included below:

FND_data$eq5d_utility_score <- 1 - (0.072*FND_data$eq5d_mobility_2 + 0.091*FND_data$eq5d_mobility_3 +

0.276*FND_data$eq5d_mobility_4 + 0.302*FND_data$eq5d_mobility_5 +

0.072*FND_data$eq5d_pers_care_2 + 0.079*FND_data$eq5d_pers_care_3 +

0.218*FND_data$eq5d_pers_care_4 + 0.301*FND_data$eq5d_pers_care_5 +

0.116*FND_data$eq5d_usual_act_2 + 0.120*FND_data$eq5d_usual_act_3 +

0.283*FND_data$eq5d_usual_act_4 + 0.283*FND_data$eq5d_usual_act_5 +

0.079*FND_data$eq5d_pain_2 + 0.089*FND_data$eq5d_pain_3 +

0.259*FND_data$eq5d_pain_4 + 0.333*FND_data$eq5d_pain_5 +

0.140*FND_data$eq5d_anx_dep_2 + 0.246*FND_data$eq5d_anx_dep_3 +

0.398*FND_data$eq5d_anx_dep_4 + 0.398*FND_data$eq5d_anx_dep_5 +

0.059*FND_data$eq5d_N5)

1. **Severity categories and score cut offs for the DASS-21**

| **Severity** | **Depression** | **Anxiety** | **Stress** |
| --- | --- | --- | --- |
| **Normal** | 0-9 | 0-7 | 0-14 |
| **Mild** | 10-13 | 8-9 | 15-18 |
| **Moderate** | 14-20 | 10-14 | 19-25 |
| **Severe** | 21-27 | 15-19 | 26-33 |
| **Extremely Severe** | 28+ | 20+ | 34+ |

Score cut-offs for the Depression, Anxiety and Stress Scale (DASS-21), defining severity categories (normal, mild, moderate, severe, extremely severe) for each subscale. Scores are doubled to align with original DASS-42 thresholds ^4^.

## Supplementary Table 1: Self report and clinician rated measures at baseline

| **Outcome Item** | **Result mean (SD)**  **(n = 101)** | **Outcome Item** | **Result mean (SD)**  **(n = 101)** |
| --- | --- | --- | --- |
| **WHODAS 2.0** | | **SF-36** | |
| *Summary score* | 50.5 (22.6) | *Physical functioning* | 47.9 (31.8) |
| *Domain 1 – Cognition* | 49.5 (24.7) | *Role limitations – physical health* | 12.6 (28.9) |
| *Domain 2 – Mobility* | 48.4 (29.1) | *Role limitations – emotional problems* | 30.4 (40.6) |
| *Domain 3 - Self-care* | 31.2 (25.5) | *Energy/Fatigue* | 26.4 (21.2) |
| *Domain 4 - Getting along with people* | 44.5 (30.7) | *Emotional wellbeing* | 46.9 (24.4) |
| *Domain 5a - Life activities – home* | 59.3 (33.6) | *Social Functioning* | 33 (29.4) |
| *Domain 5b - Life activities – work/school* | 54.4 (32) | *Pain* | 41.2 (27.8) |
| *Domain 6 – Participation* | 59.2 (23.9) | *General Health* | 33.5 (20.7) |
| *H1 - Number of days affected* | 22.1 (9.6) |  |  |
| *H2 - Days totally unable to perform duties* | 13.2 (10.8) |  |  |
| *H3 - Days needing to reduce duties* | 15.9 (11) |  |  |
|  | | | |
| **Outcome Item** | **Result mean (SD)**  **(n = 101)** | **Outcome Item** | **Result N (%)**  **(n = 101)** |
| ***EQ-5D*** | | | |
| EQ-5D Health Utility Score | 0.35 (0.37) | Mobility *(N (%))* | No problem - 36 (35.6%)  Slight - 23 (22.8%)  Moderate - 23 (22.8%)  Severe - 18 (17.8%)  Unable - 1 (1%) |
| EQ-5D VAS | 49.9 (23.2) | Personal Care *(N (%))* | No problem - 53 (52.5%)  Slight - 19 (18.8%)  Moderate - 24 (23.8%)  Severe - 5 (5%)  Unable - 0 (0%) |
|  |  | Usual Activity *(N (%))* | No problem - 18 (17.8%)  Slight - 21 (20.8%)  Moderate - 25 (24.8%)  Severe - 28 (27.7%)  Unable - 9 (8.9%) |
|  |  | Pain *(N (%))* | No problem - 17 (16.8%)  Slight - 25 (24.8%)  Moderate - 37 (36.6%)  Severe - 15 (14.9%)  Extreme - 7 (6.9%) |
|  |  | Anxiety and Depression *(N (%))* | No problem - 12 (11.9%)  Slight - 28 (27.7%)  Moderate - 31 (30.7%)  Severe - 14 (13.9%)  Extreme - 16 (15.8%) |
| **Outcome Item** | **Result mean (SD)**  **(n = 101)** | **Outcome Item** | **Result N (%)**  **(n = 101)** |
| **HoNOS score** | 10.3 (4.5) |  |  |
| **DASS-21** | | | |
| *Depression score* | 17.3 (13.4) | *Depression score categorical* | Normal - 31 (30.7%)  Mild - 15 (14.9%)  Moderate - 17 (16.8%)  Severe - 12 (11.9%)  Extremely Severe - 26 (25.7%) |
| *Anxiety score* | 16.3 (10.8) | *Anxiety score categorical* | Normal - 27 (26.7%)  Mild - 5 (5%)  Moderate - 20 (19.8%)  Severe - 11 (10.9%)  Extremely Severe - 38 (37.6%) |
| *Stress score* | 20.4 (10.4) | *Stress score categorical* | Normal - 35 (34.7%)  Mild - 15 (14.9%)  Moderate - 14 (13.9%)  Severe - 26 (25.7%)  Extremely Severe - 11 (10.9%) |
| **PHQ-15** | | | |
| PHQ-15 summary score | 15 (5.8) | PHQ-15 score categorical | Minimal - 3 (3%)  Low - 14 (13.9%)  Medium - 29 (28.7%)  High - 55 (54.5%) |
| **K10** | | | |
| K10 summary score | 29 (10.1) | K10 score categorical | Low - 10 (9.9%)  Moderate - 15 (14.9%)  High - 26 (25.7%)  Very High - 50 (49.5%) |
| **CGI-S** | | | |
| CGI-S total score | 4 (0.9) | *CGI-S score categorial* | Normal - 1 (1%)  Borderline ill - 3 (3%)  Mild - 15 (15%)  Moderate - 57 (57%)  Marked - 20 (20%)  Severe - 4 (4%)  Extreme - 0 (0%) |

**Table S1:** Self-report and clinician-rated outcome measures at baseline. SD: standard deviation; CGI-S: Clinical Global Impression – Severity scale; DASS-21: Depression Anxiety and Stress Scale (21-item); EQ-5D-5L: EQ-5D, five-level instrument; K10: Kessler Psychological Distress Scale; PHQ-15: Patient Health Questionnaire-15; WHODAS 2.0: World Health Organization Disability Assessment Schedule 2.0; SF-36: 36-Item Short Form Health Survey.

## Supplementary Table 2: Somatic symptom severity - individual items of the PHQ-15

| **PHQ-15 item (n=101)** | **Not bothered at all**  (N (%)) | **Bothered a little**  (N (%)) | **Bothered a lot**  (N (%)) |
| --- | --- | --- | --- |
| Stomach pain | 32 (31.7%) | 47 (46.5%) | 22 (21.8%) |
| Back pain | 20 (19.8%) | 40 (39.6%) | 41 (40.6%) |
| Pain in your arms, legs, or joints | 16 (15.8%) | 23 (22.8%) | 62 (61.4%) |
| Menstrual problems (n=71) | 32 (45.1%) | 24 (33.8%) | 15 (21.1%) |
| Headaches | 14 (13.9%) | 47 (46.5%) | 40 (39.6%) |
| Chest pain | 55 (54.5%) | 38 (37.6%) | 8 (7.9%) |
| Dizziness | 23 (22.8%) | 35 (34.7%) | 43 (42.6%) |
| Fainting spells | 63 (62.4%) | 22 (21.8%) | 16 (15.8%) |
| Feeling your heart pound or race | 28 (27.7%) | 49 (48.5%) | 24 (23.8%) |
| Shortness of breath | 35 (34.7%) | 48 (47.5%) | 18 (17.8%) |
| Pain or problems during sexual intercourse | 68 (67.3%) | 19 (18.8%) | 14 (13.9%) |
| Constipation, loose bowels, or diarrhea | 39 (38.6%) | 31 (30.7%) | 31 (30.7%) |
| Nausea, gas, or indigestion | 37 (36.6%) | 34 (33.7%) | 30 (29.7%) |
| Feeling tired or having low energy | 7 (6.9%) | 14 (13.9%) | 80 (79.2%) |
| Trouble sleeping | 13 (12.9%) | 23 (22.8%) | 65 (64.4%) |

**Table S2:** Individual items from the Patient Health Questionnaire, 15-item ^5^ measuring somatic symptom severity. Male participants are instructed not to answer the question on menstrual items.

## Supplementary Table 3: Comparison of baseline characteristics of male and female participants

|  | **Total (n = 105)** | **Females (n = 78)** | **Males (n = 27)** | **p-value** |
| --- | --- | --- | --- | --- |
| **Mean Age (SD) (n=105)** | 35.4 (13.3) | 33.5 (13.3) | 41 (11.9) | 0.011* |
| **Age Range (n=105)** | 17 - 66 | 17 - 66 | 26 - 63 | - |
| **Sex (M%) (n=105)** | 25.70% | 0% | 100% | - |
| **Mean socio-economic deciles (SD) (n=105)** | 7.1 (2.4) | 7.1 (2.4) | 6.9 (2.5) | 0.692 |
| **Currently working (N (%)) (n=105)** | 1 - 33 (33.7%)  2 - 52 (53.1%)  3 - 13 (13.3%) | 1 - 21 (21.4%)  2 - 38 (38.8%)  3 - 13 (13.3%) | 1 - 12 (12.2%)  2 - 14 (14.3%)  3 - 0 (0%) | 0.147 |
| **Currently studying (N (%)) (n=105)** | 1 - 11 (11.2%)  2 - 17 (17.3%)  3 - 70 (71.4%) | 1 - 10 (10.2%)  2 - 15 (15.3%)  3 - 47 (48%) | 1 - 1 (1%)  2 - 2 (2%)  3 - 23 (23.5%) | 0.333 |
| **Reason not working because of FND (N (%)) (n=105)** | 45 (42.9%) | 33 (42.3%) | 12 (44.4%) | - |
| **Mean days since last working (SD) (n=20)** | 648.6 (750.7) | 1134.3 (2200.2) | 1357.6 (1439.2) | 0.726 |
| **Mean days since last studying (SD) (n=9)** | 1096.7 (1648.9) | 897.9 (1336.4) | 1982.5 (864.8) | 0.289 |
| **Currently seeing psychologist (N (%)) (n=105)** | 50 (47.6%) | 40 (51.3%) | 10 (37%) | 0.292 |
| **Currently seeing occupation therapist (N (%)) (n=105)** | 7 (6.7%) | 6 (7.7%) | 1 (3.7%) | 0.788 |
| **Currently seeing physiotherapist (N (%)) (n=105)** | 26 (24.8%) | 20 (25.6%) | 6 (22.2%) | 0.923 |
| **Currently seeing speech pathologist (N (%)) (n=105)** | 3 (2.9%) | 3 (3.8%) | 0 (0%) | 0.716 |
| **Mean days between diagnosis and baseline (SD) (n=104)** | 356.6 (463) | 361.6 (467.1) | 342.2 (459.3) | 0.852 |
| **Mean days between referral and baseline (SD) (n=105)** | 110.4 (76.6) | 109.1 (78.1) | 114.1 (73.5) | 0.771 |
| **Mean days between diagnosis and referral (SD) (n=103)** | 251.4 (452.3) | 254 (451.5) | 243.5 (463.6) | 0.918 |
| **Non-epileptic seizures/attacks (N (%)) (n=105)** | 49 (46.7%) | 41 (52.6%) | 8 (29.6%) | 0.066 |
| **Weakness/paralysis (N (%)) (n=105)** | 48 (45.7%) | 37 (47.4%) | 11 (40.7%) | 0.706 |
| **Abnormal movement (N (%)) (n=105)** | 43 (41%) | 33 (42.3%) | 10 (37%) | 0.8 |
| **Speech symptom (N (%)) (n=105)** | 20 (19%) | 14 (17.9%) | 6 (22.2%) | 0.839 |
| **Sensory disturbance (N (%)) (n=105)** | 26 (24.8%) | 17 (21.8%) | 9 (33.3%) | 0.348 |
| **Other (N (%)) (n=105)** | 19 (18.1%) | 13 (16.7%) | 6 (22.2%) | 0.722 |
| **Fatigue (N (%)) (n=105)** | 48 (45.7%) | 37 (47.4%) | 11 (40.7%) | 0.706 |
| **Cognitive Symptoms (N (%)) (n=105)** | 41 (39%) | 29 (37.2%) | 12 (44.4%) | 0.661 |
| **Gastrointestinal symptoms (N (%)) (n=105)** | 6 (5.7%) | 6 (7.7%) | 0 (0%) | 0.316 |
| **Other (N (%)) (n=105)** | 15 (14.3%) | 10 (12.8%) | 5 (18.5%) | 0.682 |
| **Number of presentations to emergency (N (%)) (n=105)** | 0 - 34 (32.4%)  1 - 28 (26.7%)  2 - 22 (21%)  3+ - 21 (20%) | 0 - 18 (23.1%)  1 - 25 (32.1%)  2 - 16 (20.5%)  3+ - 19 (24.4%) | 0 - 16 (59.3%)  1 - 3 (11.1%)  2 - 6 (22.2%)  3+ - 2 (7.4%) | 0.003* |
| **Number of admissions to hospitals (N (%)) (n=105)** | 0 - 41 (39%)  1 - 38 (36.2%)  2 - 19 (18.1%)  3+ - 7 (6.7%) | 0 - 25 (32.1%)  1 - 31 (39.7%)  2 - 16 (20.5%)  3+ - 6 (7.7%) | 0 - 16 (59.3%)  1 - 7 (25.9%)  2 - 3 (11.1%)  3+ - 1 (3.7%) | 0.097 |

**Table S3:** Comparing baseline characteristics of males and females in this cohort of patients with functional neurological disorders (FND). For ‘currently working’ and ‘currently studying’, 1=yes, 2=no, 3=not applicable. * p-value is statistically significant.

## Supplementary Table 4: Comparison of baseline outcomes of males and females

|  | **Total (n = 101)** | **Females (n = 74)** | | **Males (n = 27)** | | **p-values** | |  |
| --- | --- | --- | --- | --- | --- | --- | --- | --- |
| ***WHODAS 2.0*** |  | |  | |  | |  | |
| ***Summary score (mean (SD))*** | 50.5 (22.6) | | 51.1 (21.7) | | 48.9 (25.1) | | 0.67 | |
| ***Domain 1 – Cognition (mean (SD))*** | 49.5 (24.7) | | 50.3 (24.1) | | 47.2 (26.6) | | 0.586 | |
| ***Domain 2 – Mobility (mean (SD))*** | 48.4 (29.1) | | 48.5 (28.2) | | 48.1 (32) | | 0.96 | |
| ***Domain 3 - Self-care (mean (SD))*** | 31.2 (25.5) | | 31.6 (23.6) | | 30 (30.4) | | 0.779 | |
| ***Domain 4 - Getting along with people (mean (SD))*** | 44.5 (30.7) | | 43.9 (31.2) | | 46 (29.9) | | 0.766 | |
| ***Domain 5a - Life activities – home (mean (SD))*** | 59.3 (33.6) | | 59.2 (33.6) | | 59.6 (34) | | 0.954 | |
| ***Domain 5b - Life activities – work/school (mean (SD))*** | 54.4 (32) | | 56.4 (31.7) | | 50 (33.1) | | 0.51 | |
| ***Domain 6 – Participation (mean (SD))*** | 59.2 (23.9) | | 60.5 (23.8) | | 55.6 (24.5) | | 0.358 | |
| ***H1 - Number of days affected (mean (SD))*** | 22.1 (9.6) | | 21.9 (9.7) | | 22.8 (9.6) | | 0.676 | |
| ***H2 - Days totally unable to perform duties (mean (SD))*** | 13.2 (10.8) | | 12.7 (10) | | 14.7 (12.7) | | 0.423 | |
| ***H3 - Days needing to reduce duties (mean (SD))*** | 15.9 (11) | | 15.8 (10.7) | | 16.1 (11.8) | | 0.887 | |
| **DASS-21** |  | |  | |  | |  | |
| ***Depression score (mean (SD))*** | 17.3 (13.4) | | 17.2 (13.2) | | 17.6 (14.2) | | 0.892 | |
| ***Anxiety score (mean (SD))*** | 16.3 (10.8) | | 16.4 (10.6) | | 16.2 (11.6) | | 0.967 | |
| ***Stress score (mean (SD))*** | 20.4 (10.4) | | 20.6 (10.2) | | 20 (10.8) | | 0.794 | |
| **Mean PHQ-15 score (SD)** | 15 (5.8) | | 15.4 (5.9) | | 13.7 (5.7) | | 0.189 | |
| **PHQ-15 score categorical (N (%))** | Minimal - 3 (3%)  Low - 14 (13.9%)  Medium - 29 (28.7%)  High - 55 (54.5%) | | Minimal - 2 (2.7%)  Low - 11 (14.9%)  Medium - 15 (20.3%)  High - 46 (62.2%) | | Minimal - 1 (3.7%)  Low - 3 (11.1%)  Medium - 14 (51.9%)  High - 9 (33.3%) | | - | |
| **Mean K10 (SD)** | 29 (10.1) | | 29 (10) | | 29 (10.8) | | 0.987 | |
| **K10 score categorical (N (%))** | Low - 10 (9.9%)  Moderate - 15 (14.9%)  High - 26 (25.7%)  Very High - 50 (49.5%) | | Low - 9 (12.2%)  Moderate - 8 (10.8%)  High - 19 (25.7%)  Very High - 38 (51.4%) | | Low - 1 (3.7%)  Moderate - 7 (25.9%)  High - 7 (25.9%)  Very High - 12 (44.4%) | | - | |
| **EQ-5D-5L** |  | |  | |  | |  | |
| **Mobility *(N (%))*** | No problem - 36 (35.6%)  Slight - 23 (22.8%)  Moderate - 23 (22.8%)  Severe - 18 (17.8%)  Unable - 1 (1%) | | No problem - 29 (39.2%)  Slight - 18 (24.3%)  Moderate - 13 (17.6%)  Severe - 13 (17.6%)  Unable - 1 (1.4%) | | No problem - 7 (25.9%)  Slight - 5 (18.5%)  Moderate - 10 (37%)  Severe - 5 (18.5%)  Unable - 0 (0%) | | 0.241 | |
| **Personal Care *(N (%))*** | No problem - 53 (52.5%)  Slight - 19 (18.8%)  Moderate - 24 (23.8%)  Severe - 5 (5%)  Unable - 0 (0%) | | No problem - 38 (51.4%)  Slight - 17 (23%)  Moderate - 16 (21.6%)  Severe - 3 (4.1%)  Unable - 0 (0%) | | No problem - 15 (55.6%)  Slight - 2 (7.4%)  Moderate - 8 (29.6%)  Severe - 2 (7.4%)  Unable - 0 (0%) | | 0.631 | |
| **Usual Activity *(N (%))*** | No problem - 18 (17.8%)  Slight - 21 (20.8%)  Moderate - 25 (24.8%)  Severe - 28 (27.7%)  Unable - 9 (8.9%) | | No problem - 15 (20.3%)  Slight - 17 (23%)  Moderate - 17 (23%)  Severe - 19 (25.7%)  Unable - 6 (8.1%) | | No problem - 3 (11.1%)  Slight - 4 (14.8%)  Moderate - 8 (29.6%)  Severe - 9 (33.3%)  Unable - 3 (11.1%) | | 0.154 | |
| **Pain *(N (%))*** | No problem - 17 (16.8%)  Slight - 25 (24.8%)  Moderate - 37 (36.6%)  Severe - 15 (14.9%)  Extreme - 7 (6.9%) | | No problem - 15 (20.3%)  Slight - 15 (20.3%)  Moderate - 30 (40.5%)  Severe - 11 (14.9%)  Extreme - 3 (4.1%) | | No problem - 2 (7.4%)  Slight - 10 (37%)  Moderate - 7 (25.9%)  Severe - 4 (14.8%)  Extreme - 4 (14.8%) | | 0.232 | |
| **Anxiety and Depression *(N (%))*** | No problem - 12 (11.9%)  Slight - 28 (27.7%)  Moderate - 31 (30.7%)  Severe - 14 (13.9%)  Extreme - 16 (15.8%) | | No problem - 7 (9.5%)  Slight - 23 (31.1%)  Moderate - 22 (29.7%)  Severe - 10 (13.5%)  Extreme - 12 (16.2%) | | No problem - 5 (18.5%)  Slight - 5 (18.5%)  Moderate - 9 (33.3%)  Severe - 4 (14.8%)  Extreme - 4 (14.8%) | | 0.802 | |
| **Mean EQ-5D Health Utility Score (SD)** | 0.35 (0.37) | | 0.37 (0.34) | | 0.29 (0.42) | | 0.024* | |
| **EQVAS - self-rated health today (mean (SD))** | 49.9 (23.2) | | 53 (22.1) | | 41.3 (24.4) | | 0.335 | |
| **SF-36** |  | |  | |  | |  | |
| ***Physical functioning* (mean (SD))** | 47.9 (31.8) | | 47.8 (32.1) | | 48 (31.4) | | 0.986 | |
| ***Role limitations – physical health* (mean (SD))** | 12.6 (28.9) | | 12.2 (28.7) | | 13.9 (29.7) | | 0.792 | |
| ***Role limitations – emotional problems* (mean (SD))** | 30.4 (40.6) | | 31.1 (40.3) | | 28.4 (42.1) | | 0.77 | |
| ***Energy/Fatigue* (mean (SD))** | 26.4 (21.2) | | 24.9 (20.8) | | 30.7 (21.9) | | 0.219 | |
| ***Emotional wellbeing* (mean (SD))** | 46.9 (24.4) | | 45.9 (24.4) | | 49.6 (24.6) | | 0.498 | |
| ***Social Functioning* (mean (SD))** | 33 (29.4) | | 32.9 (28.9) | | 33.3 (31.4) | | 0.953 | |
| ***Pain* (mean (SD))** | 41.2 (27.8) | | 41.2 (27.4) | | 41.2 (29.3) | | 0.994 | |
| ***General Health* (mean (SD))** | 33.5 (20.7) | | 33.2 (21.3) | | 34.3 (19.4) | | 0.829 | |
| **Mean CGI-S (SD)** | 4 (0.9) | | 4.1 (0.8) | | 4 (0.9) | | 0.782 | |
| ***CGI-S categorial (N (%))*** | Normal - 1 (1%)  Borderline ill - 3 (3%)  Mild - 15 (15%)  Moderate - 57 (57%)  Marked - 20 (20%)  Severe - 4 (4%)  Extreme - 0 (0%) | | Normal - 1 (1.4%)  Borderline ill - 1 (1.4%)  Mild - 12 (16.2%)  Moderate - 42 (56.8%)  Marked - 15 (20.3%)  Severe - 3 (4.1%)  Extreme - 0 (0%) | | Normal - 0 (0%)  Borderline ill - 2 (7.7%)  Mild - 3 (11.5%)  Moderate - 15 (57.7%)  Marked - 5 (19.2%)  Severe - 1 (3.8%)  Extreme - 0 (0%) | | - | |
| **Mean HoNOS Score (SD)** | 10.3 (4.5) | | 10.4 (4.4) | | 9.9 (4.8) | | 0.604 | |

**Table S4:** Comparing baseline outcomes of males and females in this cohort of patients with functional neurological disorders (FND). CGI-S: Clinical Global Impression – Severity scale; DASS-21: Depression Anxiety and Stress Scale (21-item); EQ-5D-5L: EQ-5D, five-level instrument; K10: Kessler Psychological Distress Scale; PHQ-15: Patient Health Questionnaire-15; WHODAS 2.0: World Health Organization Disability Assessment Schedule 2.0; SF-36: 36-Item Short Form Health Survey. HoNOS: Health of the Nation Outcome Scale.

## Supplementary Table 5: Comparison of baseline characteristics of the three age tertiles

|  | **Total** | **17 -25 (n = 29)** | **26 - 40 (n = 42)** | **41+ (n = 34)** | **p-value** |
| --- | --- | --- | --- | --- | --- |
| **Mean Age (SD) (n=105)** | 35.4 (13.3) | 20.7 (2) | 32.4 (4.5) | 51.8 (7.2) | <0.0001 |
| **Age Range (n=105)** | 17 - 66 | 17 - 24 | 26 - 40 | 41 - 66 | - |
| **Sex (M%) (n=105)** | 25.70% | 0% | 35.70% | 35.30% | 0.001 |
| **Mean Socio-economic Deciles (SD) (n=105)** | 7.1 (2.4) | 6.9 (2.9) | 7.1 (2.2) | 7.2 (2.2) | 0.657 |
| **Currently Working (N (%)) (n=105)** | 1 - 33 (33.7%)  2 - 52 (53.1%)  3 - 13 (13.3%) | 1 - 9 (9.2%)  2 - 10 (10.2%)  3 - 9 (9.2%) | 1 - 16 (16.3%)  2 - 22 (22.4%)  3 - 2 (2%) | 1 - 8 (8.2%)  2 - 20 (20.4%)  3 - 2 (2%) | 0.396 |
| **Currently Studying (N (%)) (n=105)** | 1 - 11 (11.2%)  2 - 17 (17.3%)  3 - 70 (71.4%) | 1 - 10 (10.2%)  2 - 11 (11.2%)  3 - 7 (7.1%) | 1 - 1 (1%)  2 - 5 (5.1%)  3 - 34 (34.7%) | 1 - 0 (0%)  2 - 1 (1%)  3 - 29 (29.6%) | <0.0001 |
| **Reason not working because of FND (N (%)) (n=105)** | 45 (42.9%) | 10 (34.5%) | 20 (47.6%) | 15 (44.1%) | - |
| **Mean days since last working (SD) (n=20)** | 1194.4 (2012.4) | 226.8 (142.6) | 996 (1329.4) | 1896.6 (2801.8) | 0.025 |
| **Mean days since last studying (SD) (n=9)** | 1025.5 (1318.8) | 595.8 (694.2) | 1901.8 (2062.6) | 1371 (NA) | 0.123 |
| **Currently seeing psychologist (N (%)) (n=105)** | 50 (47.6%) | 15 (51.7%) | 21 (50%) | 14 (41.2%) | 0.651 |
| **Currently seeing occupation therapist (N (%)) (n=105)** | 7 (6.7%) | 3 (10.3%) | 2 (4.8%) | 2 (5.9%) | 0.635 |
| **Currently seeing physiotherapist (N (%)) (n=105)** | 26 (24.8%) | 8 (27.6%) | 7 (16.7%) | 11 (32.4%) | 0.265 |
| **Currently seeing speech pathologist (N (%)) (n=105)** | 3 (2.9%) | 1 (3.4%) | 0 (0%) | 2 (5.9%) | 0.302 |
| **Mean days between diagnosis and baseline (SD) (n=104)** | 356.6 (463) | 326.1 (342.1) | 293.2 (363.7) | 460 (626.1) | 0.231 |
| **Mean days between referral and baseline (SD) (n=105)** | 110.4 (76.6) | 110.1 (72.5) | 108 (75.5) | 113.5 (83.3) | 0.852 |
| **Mean days between diagnosis and referral (SD) (n=103)** | 251.4 (452.3) | 220 (343.4) | 193.9 (360.4) | 346.5 (603.5) | 0.249 |
| **Non-epileptic seizures/attacks (N (%)) (n=105)** | 49 (46.7%) | 20 (69%) | 19 (45.2%) | 10 (29.4%) | 0.007 |
| **Weakness/paralysis (N (%)) (n=105)** | 48 (45.7%) | 13 (44.8%) | 20 (47.6%) | 15 (44.1%) | 0.949 |
| **Abnormal movement (N (%)) (n=105)** | 43 (41%) | 9 (31%) | 16 (38.1%) | 18 (52.9%) | 0.188 |
| **Speech symptom (N (%)) (n=105)** | 20 (19%) | 2 (6.9%) | 8 (19%) | 10 (29.4%) | 0.076 |
| **Sensory disturbance (N (%)) (n=105)** | 26 (24.8%) | 4 (13.8%) | 15 (35.7%) | 7 (20.6%) | 0.087 |
| **Other (N (%)) (n=105)** | 19 (18.1%) | 3 (10.3%) | 8 (19%) | 8 (23.5%) | 0.391 |
| **Fatigue (N (%)) (n=105)** | 48 (45.7%) | 12 (41.4%) | 20 (47.6%) | 16 (47.1%) | 0.858 |
| **Cognitive Symptoms (N (%)) (n=105)** | 41 (39%) | 7 (24.1%) | 15 (35.7%) | 19 (55.9%) | 0.031 |
| **Gastrointestinal symptoms (N (%)) (n=105)** | 6 (5.7%) | 2 (6.9%) | 2 (4.8%) | 2 (5.9%) | 0.929 |
| **Other (N (%)) (n=105)** | 15 (14.3%) | 1 (3.4%) | 9 (21.4%) | 5 (14.7%) | 0.103 |
| **Number of presentations to emergency (N (%)) (n=105)** | 0 - 34 (32.4%)  1 - 28 (26.7%)  2 - 22 (21%)  3+ - 21 (20%) | 0 - 2 (6.9%)  1 - 12 (41.4%)  2 - 5 (17.2%)  3+ - 10 (34.5%) | 0 - 14 (33.3%)  1 - 8 (19%)  2 - 10 (23.8%)  3+ - 10 (23.8%) | 0 - 18 (52.9%)  1 - 8 (23.5%)  2 - 7 (20.6%)  3+ - 1 (2.9%) | 0.001 |
| **Number of admissions to hospitals (N (%)) (n=105)** | 0 - 41 (39%)  1 - 38 (36.2%)  2 - 19 (18.1%)  3+ - 7 (6.7%) | 0 - 11 (37.9%)  1 - 11 (37.9%)  2 - 5 (17.2%)  3+ - 2 (6.9%) | 0 - 14 (33.3%)  1 - 16 (38.1%)  2 - 8 (19%)  3+ - 4 (9.5%) | 0 - 16 (47.1%)  1 - 11 (32.4%)  2 - 6 (17.6%)  3+ - 1 (2.9%) | 0.881 |

**Table S5:** Comparing baseline characteristics of the age tertiles (17–25, 26–40, 41+)

in this cohort of patients with functional neurological disorders (FND). For ‘currently working’ and ‘currently studying’, 1=yes, 2=no, 3=not applicable.

## Supplementary Table 6: Comparison of baseline outcomes of the age tertiles

|  | **Total (n = 101)** | **17 - 25 (n = 28)** | **26 - 40 (n = 41)** | **41+ (n = 32)** | **p-value** |
| --- | --- | --- | --- | --- | --- |
| ***WHODAS 2.0*** |  |  |  |  |  |
| ***Summary score (mean (SD))*** | 50.5 (22.6) | 49.5 (20.9) | 52.1 (23.7) | 49.1 (23.1) | 0.934 |
| ***Domain 1 – Cognition (mean (SD))*** | 49.5 (24.7) | 49.6 (19.7) | 48.8 (25.7) | 50.2 (27.9) | 0.931 |
| ***Domain 2 – Mobility (mean (SD))*** | 48.4 (29.1) | 48.2 (25.7) | 50.2 (30.3) | 46.3 (31.1) | 0.783 |
| ***Domain 3 - Self-care (mean (SD))*** | 31.2 (25.5) | 31.1 (22.8) | 34.4 (26.7) | 27.2 (26.2) | 0.529 |
| ***Domain 4 - Getting along with people (mean (SD))*** | 44.5 (30.7) | 46.7 (30.3) | 47 (33) | 39.3 (28.3) | 0.34 |
| ***Domain 5a - Life activities – home (mean (SD))*** | 59.3 (33.6) | 53.2 (32.7) | 61.7 (35.3) | 61.6 (32.4) | 0.352 |
| ***Domain 5b - Life activities – work/ school (mean (SD))*** | 54.4 (32) | 49.1 (24.4) | 57.1 (33.8) | 56.1 (37.5) | 0.538 |
| ***Domain 6 – Participation (mean (SD))*** | 59.2 (23.9) | 57.7 (23.9) | 62.3 (23.1) | 56.5 (25.4) | 0.809 |
| ***H1 - Number of days affected (mean (SD))*** | 22.1 (9.6) | 20.8 (8.6) | 22 (10.3) | 23.4 (9.8) | 0.294 |
| ***H2 - Days totally unable to perform duties (mean (SD))*** | 13.2 (10.8) | 10.8 (8.8) | 13 (10.5) | 15.8 (12.3) | 0.07 |
| ***H3 - Days needing to reduce duties (mean (SD))*** | 15.9 (11) | 16.4 (10.4) | 14.2 (10.6) | 17.5 (11.9) | 0.643 |
| **DASS-21** |  |  |  |  |  |
| ***Depression score (mean (SD))*** | 17.3 (13.4) | 18.2 (13.8) | 17.6 (13.0) | 16.2 (13.8) | 0.57 |
| ***Anxiety score (mean (SD))*** | 16.3 (10.8) | 18.8 (11.4) | 15.6 (9.8) | 15.0 (11.6) | 0.179 |
| ***Stress score (mean (SD))*** | 20.4 (10.4) | 21.6 (10.4) | 21.2 (10.2) | 18.4 (10.6) | 0.226 |
| **Mean PHQ-15 score (SD)** | 15 (5.8) | 17.4 (5.2) | 14.8 (6.4) | 13.1 (5) | 0.005 |
| **PHQ-15 score categorical (N (%))** | Minimal - 3 (3%)  Low - 14 (13.9%)  Medium - 29 (28.7%)  High - 55 (54.5%) | Minimal - 1 (3.6%)  Low - 1 (3.6%)  Medium - 4 (14.3%)  High - 22 (78.6%) | Minimal - 2 (4.9%)  Low - 7 (17.1%)  Medium - 10 (24.4%)  High - 22 (53.7%) | Minimal - 0 (0%)  Low - 6 (18.8%)  Medium - 15 (46.9%)  High - 11 (34.4%) | - |
| **Mean K10 (SD)** | 29 (10.1) | 31.1 (10.2) | 28.5 (9.8) | 27.8 (10.5) | 0.227 |
| **K10 score categorical (N (%))** | Low - 10 (9.9%)  Moderate - 15 (14.9%)  High - 26 (25.7%)  Very High - 50 (49.5%) | Low - 3 (10.7%)  Moderate - 2 (7.1%)  High - 7 (25%)  Very High - 16 (57.1%) | Low - 3 (7.3%)  Moderate - 7 (17.1%)  High - 12 (29.3%) Very High - 19 (46.3%) | Low - 4 (12.5%)  Moderate - 6 (18.8%)  High - 7 (21.9%)  Very High - 15 (46.9%) | - |
| **EQ-5D-5L** |  |  |  |  |  |
| **Mobility *(N (%))*** | No problem - 36 (35.6%)  Slight - 23 (22.8%)  Moderate - 23 (22.8%)  Severe - 18 (17.8%)  Unable - 1 (1%) | No problem - 9 (32.1%)  Slight - 11 (39.3%)  Moderate - 5 (17.9%)  Severe - 3 (10.7%)  Unable - 0 (0%) | No problem - 17 (41.5%)  Slight - 8 (19.5%) Moderate - 8 (19.5%) Severe - 8 (19.5%) Unable - 0 (0%) | No problem - 10 (31.2%)  Slight - 4 (12.5%)  Moderate - 10 (31.2%)  Severe - 7 (21.9%)  Unable - 1 (3.1%) | 0.118 |
| **Personal Care *(N (%))*** | No problem - 53 (52.5%)  Slight - 19 (18.8%)  Moderate - 24 (23.8%)  Severe - 5 (5%)  Unable - 0 (0%) | No problem - 15 (53.6%)  Slight - 7 (25%)  Moderate - 5 (17.9%)  Severe - 1 (3.6%)  Unable - 0 (0%) | No problem - 22 (53.7%)  Slight - 6 (14.6%)  Moderate - 12 (29.3%)  Severe - 1 (2.4%)  Unable - 0 (0%) | No problem - 16 (50%)  Slight - 6 (18.8%)  Moderate - 7 (21.9%)  Severe - 3 (9.4%)  Unable - 0 (0%) | 0.444 |
| **Usual Activity *(N (%))*** | No problem - 18 (17.8%)  Slight - 21 (20.8%)  Moderate - 25 (24.8%)  Severe - 28 (27.7%)  Unable - 9 (8.9%) | No problem - 4 (14.3%)  Slight - 10 (35.7%)  Moderate - 9 (32.1%)  Severe - 5 (17.9%)  Unable - 0 (0%) | No problem - 9 (22%)  Slight - 5 (12.2%)  Moderate - 8 (19.5%)  Severe - 15 (36.6%) Unable - 4 (9.8%) | No problem - 5 (15.6%)  Slight - 6 (18.8%)  Moderate - 8 (25%)  Severe - 8 (25%)  Unable - 5 (15.6%) | 0.11 |
| **Pain *(N (%))*** | No problem - 17 (16.8%)  Slight - 25 (24.8%)  Moderate - 37 (36.6%)  Severe - 15 (14.9%)  Extreme - 7 (6.9%) | No problem - 4 (14.3%)  Slight - 5 (17.9%)  Moderate - 14 (50%)  Severe - 5 (17.9%)  Extreme - 0 (0%) | No problem - 7 (17.1%)  Slight - 11 (26.8%)  Moderate - 11 (26.8%)  Severe - 7 (17.1%) Extreme - 5 (12.2%) | No problem - 6 (18.8%)  Slight - 9 (28.1%)  Moderate - 12 (37.5%)  Severe - 3 (9.4%)  Extreme - 2 (6.2%) | 0.584 |
| **Anxiety and Depression *(N (%))*** | No problem - 12 (11.9%)  Slight - 28 (27.7%)  Moderate - 31 (30.7%)  Severe - 14 (13.9%)  Extreme - 16 (15.8%) | No problem - 3 (10.7%)  Slight - 6 (21.4%)  Moderate - 9 (32.1%)  Severe - 4 (14.3%)  Extreme - 6 (21.4%) | No problem - 4 (9.8%)  Slight - 11 (26.8%)  Moderate - 13 (31.7%)  Severe - 6 (14.6%)  Extreme - 7 (17.1%) | No problem - 5 (15.6%)  Slight - 11 (34.4%)  Moderate - 9 (28.1%)  Severe - 4 (12.5%)  Extreme - 3 (9.4%) | 0.124 |
| **Mean EQ-5D Health Utility Score (SD)** | 0.35 (0.37) | 0.39 (0.3) | 0.31 (0.39) | 0.35 (0.39) | 0.748 |
| **EQVAS - self-rated health (mean (SD))** | 49.9 (23.2) | 55.4 (20.1) | 48.9 (26.3) | 46.4 (21.3) | 0.142 |
| **SF-36** |  |  |  |  |  |
| ***Physical functioning* (mean (SD))** | 47.9 (31.8) | 50.7 (31.3) | 48.2 (32.1) | 45 (32.6) | 0.488 |
| ***Role limitations – physical health* (mean (SD))** | 12.6 (28.9) | 8.9 (26.5) | 15.2 (33) | 12.5 (25.4) | 0.658 |
| ***Role limitations – emotional problems* (mean (SD))** | 30.4 (40.6) | 23.8 (36.1) | 35 (42.8) | 30.2 (41.8) | 0.572 |
| ***Energy/Fatigue* (mean (SD))** | 26.4 (21.2) | 21.2 (17.1) | 24.9 (23.9) | 33 (19.5) | 0.03 |
| ***Emotional wellbeing* (mean (SD))** | 46.9 (24.4) | 42.6 (25.3) | 46.4 (23) | 51.2 (25.2) | 0.168 |
| ***Social Functioning* (mean (SD))** | 33 (29.4) | 37.1 (28.6) | 29.6 (30.6) | 34 (29) | 0.72 |
| ***Pain* (mean (SD))** | 41.2 (27.8) | 36.4 (27) | 41.4 (29.1) | 45.2 (27) | 0.224 |
| ***General Health* (mean (SD))** | 33.5 (20.7) | 27.5 (18.4) | 32.2 (20.1) | 40.5 (22) | 0.014 |
| **Mean CGI-S (SD)** | 4 (0.9) | 4.1 (0.6) | 4 (1) | 4 (0.8) | 0.636 |
| ***CGI-S categorial (N (%))*** | Normal - 1 (1%)  Borderline ill - 3 (3%)  Mild - 15 (15%)  Moderate - 57 (57%)  Marked - 20 (20%)  Severe - 4 (4%)  Extreme - 0 (0%) | Normal - 0 (0%)  Borderline ill - 0 (0%)  Mild - 3 (10.7%)  Moderate - 20 (71.4%)  Marked - 4 (14.3%)  Severe - 1 (3.6%)  Extreme - 0 (0%) | Normal - 1 (2.4%)  Borderline ill - 3 (7.3%)  Mild - 4 (9.8%)  Moderate - 21 (51.2%)  Marked - 10 (24.4%)  Severe - 2 (4.9%)  Extreme - 0 (0%) | Normal - 0 (0%)  Borderline ill - 0 (0%)  Mild - 8 (25.8%)  Moderate - 16 (51.6%)  Marked - 6 (19.4%)  Severe - 1 (3.2%)  Extreme - 0 (0%) | - |
| **Mean HoNOS Score (SD)** | 10.3 (4.5) | 10 (4.2) | 10.1 (4.9) | 10.8 (4.2) | 0.451 |

**Table S6:** Comparing baseline outcomes of age tertiles in this cohort of patients with functional neurological disorders (FND).

CGI-S: Clinical Global Impression – Severity scale; DASS-21: Depression Anxiety and Stress Scale (21-item); EQ-5D-5L: EQ-5D, five-level instrument; K10: Kessler Psychological Distress Scale; PHQ-15: Patient Health Questionnaire-15; WHODAS 2.0: World Health Organization Disability Assessment Schedule 2.0; SF-36: 36-Item Short Form Health Survey. HoNOS: Health of the Nation Outcome Scale.

## Supplementary References

1. Üstün T. Measuring Health and Disability: Manual for WHO Disability Assessment Schedule - WHODAS 2.0. World Health Organization; 2010.

2. Redwood L, Currow D, Kochovska S, Thomas SJ. Australian population norms for health-related quality of life measured using the EQ-5D-5L, and relationships with sociodemographic characteristics. Qual Life Res 2024;33(3):721-33.

3. Norman R, Mulhern B, Lancsar E, Lorgelly P, Ratcliffe J, Street D, et al. The Use of a Discrete Choice Experiment Including Both Duration and Dead for the Development of an EQ-5D-5L Value Set for Australia. Pharmacoeconomics 2023;41(4):427-38.

4. Lovibond SH, Lovibond PF. Manual for the depression anxiety stress scales. Sydney Psychology Foundation; 1995.

5. Kroenke K, Spitzer RL, Williams JB. The PHQ-15: validity of a new measure for evaluating the severity of somatic symptoms. Psychosom Med 2002;64(2):258-66.
